# The Prebiotic Effects of an Extract with Antioxidant Properties from *Morus alba* L. Contribute to Ameliorate High-Fat Diet-Induced Obesity in Mice

**DOI:** 10.3390/antiox12040978

**Published:** 2023-04-21

**Authors:** María Jesús Rodríguez-Sojo, Antonio Jesús Ruiz-Malagón, Laura Hidalgo-García, Jose Alberto Molina-Tijeras, Patricia Diez-Echave, Laura López-Escanez, Lucrezia Rosati, Elena González-Lozano, Laura Cenis-Cifuentes, Jorge García-García, Federico García, Iñaki Robles-Vera, Miguel Romero, Juan Duarte, José Luis Cenis, Antonio Abel Lozano-Pérez, Julio Gálvez, María Elena Rodríguez-Cabezas, Alba Rodríguez-Nogales

**Affiliations:** 1 Department of Pharmacology, Center for Biomedical Research (CIBM), University of Granada, 18071 Granada, Spain; mjrodriguez@ugr.es (M.J.R.-S.);; 2 Instituto de Investigación Biosanitaria de Granada (ibs. GRANADA), 18012 Granada, Spain; 3 Department of Medicine and Surgery, University of Perugia, 06132 Perugia, Italy; 4 Centro de Salud Floridablanca, 30002 Murcia, Spain; 5 Servicio Microbiología, Hospital Universitario Clínico San Cecilio, 18100 Granada, Spain; 6 Centro de Investigación Biomédica en Red de Enfermedades Infecciosas (CIBER-INFECC), Instituto Salud Carlos III, 28029 Madrid, Spain; 7 Centro de Investigación Biomédica en Red de Enfermedades Cardiovasculares (CIBER-CV), Instituto Salud Carlos III, 28029 Madrid, Spain; 8 Instituto Murciano de Investigación y Desarrollo Agrario y Alimentario, 30150 Murcia, Spain; 9 Instituto Murciano de Investigación Biosanitaria (IMIB)-Arrixaca, 30120 Murcia, Spain; 10 Centro de Investigación Biomédica en Red de Enfermedades Hepáticas y Digestivas (CIBER-EHD), Instituto Salud Carlos III, 28029 Madrid, Spain

**Keywords:** *Morus alba* L., obesity, microbiota, antioxidant

## Abstract

Obesity is a global health issue, in which modifications in gut microbiota composition have a key role. Different therapeutic strategies are being developed in combination with diet and exercise, including the use of plant extracts, such as those obtained from *Morus alba* L. leaves. Recent studies have revealed their anti-inflammatory and antioxidant properties. The aim of the present work was to evaluate whether the beneficial effects of *M. alba* L. leaf extract in high-fat diet-induced obesity in mice is correlated with its impact on gut microbiota. The extract reduced body weight gain and attenuated lipid accumulation, as well as increased glucose sensitivity. These effects were associated with an amelioration of the obesity-associated inflammatory status, most probably due to the described antioxidant properties of the extract. Moreover, *M. alba* L. leaf extract mitigated gut dysbiosis, which was evidenced by the restoration of the *Firmicutes*/*Bacteroidota* ratio and the decrease in plasma lipopolysaccharide (LPS) levels. Specifically, the extract administration reduced *Alistipes* and increased *Faecalibaculum* abundance, these effects being correlated with the beneficial effects exerted by the extract on the obesity-associated inflammation. In conclusion, anti-obesogenic effects of *M. alba* L. leaf extract may be mediated through the amelioration of gut dysbiosis.

## 1. Introduction

Obesity is one of the most serious and prevalent non-communicable diseases of the 21st century, constituting a global public health issue. In fact, according to the last report from the World Health Organization, it has been estimated that more than 1.9 billion adults (>18 years old) are overweight [1]. Consistently, obesity is becoming a worldwide epidemic and presents major therapeutic challenges due to its associated high risk for developing certain pathologies, including type 2 diabetes, cardiovascular disease, cancer and depression [2]. Several strategies have been developed to face-off obesity and overweight status, especially through exercise training or reducing energy intake [3]. However, these options usually require prolonged periods of time and an important dedication until results are evident, and this can lead to a lack of long-term efficacy. This has promoted the use of alternative strategies, including pharmacological treatments or, in extreme cases, surgery.

However, these alternatives may present problems with their long-term usage and effectiveness and are not as safe as diet and exercise [4,5]. In consequence, there is a clear necessity to look for alternative and/or complementary treatments in the prevention and/or treatment of obesity, which must show efficacy and safety. In this context, plant extracts containing different active compounds with antioxidant properties can be considered promising candidates [6].

This could be the case of *Morus alba* L., also known as white mulberry, which is a medium-size tree to which numerous nutritional and medicinal properties have been attributed. Since ancient times, the leaves of *M. alba* L. have been widely used in traditional Chinese medicine as a treatment against fever, constipation and diabetes [7,8]. The latter has been supported by different studies, both in humans and in preclinical models, which have demonstrated significant improvements in both glucose and lipid metabolism by effectively reducing plasma glucose levels and ameliorating the hyperglycemia-associated damage in different target organs [9,10]. In fact, the presence of flavonoids (such as quercetin, rutin or apigenin), phenolic acids (like ferulic, chlorogenic and protocatechuic acids) as well as alkaloids, including 1–deoxynojirimycin, has been reported; these have been proposed to be responsible for the beneficial effects exerted by this extract in metabolic disorders due to their antioxidant and/or anti-inflammatory properties [11,12].

Interestingly, polyphenolic-enriched plant extracts with antioxidant properties have been considered as prebiotics, due to their ability to selectively promote the growth of beneficial bacteria in the gut [13]. This is relevant when considering that obesity-associated comorbidities, including metabolic and cardiovascular disorders, have been clearly related to dysbiosis [14], which has been associated with an impairment of gut permeability and energy homeostasis [15]. In consequence, the amelioration of gut dysbiosis can be considered as an appropriate strategy for the management of obesity and its related diseases.

In a previous study, the beneficial effects of different extracts of *M. alba* L. leaves were reported in an experimental model of obesity in mice fed a high-fat diet to be associated with their antioxidant and anti-inflammatory activities [10]. Phytochemical compounds such as protocatechuic acid and flavonoids including quercetin and their derivatives have been reported to be present in different varieties of *M. alba* L., but not in the same abundance [10]. Moreover, these phytochemicals may be involved in the beneficial effects exerted by *M. alba* L. against obesity and its associated disorders [10]. Additionally, these isolated compounds have been reported to exert prebiotic, anti-inflammatory and anti-obesogenic properties [16,17,18]. In the present study, the role of the prebiotic properties of an extract obtained from the leaves of *M. alba* L. was investigated in a murine model of high-fat diet-induced obesity, comparing its effects to those of metformin and correlating them with its impact on the obesity-associated inflammation and oxidative stress that characterize this condition [19].

## 2. Materials and Methods

### 2.1. Plant Extract and Reagents

The extract was obtained as previously described [10] from the leaves of *Morus alba* L., genotype BGMU 050 10009 (*Italia*) from Mulberry Germplasm Bank of the Sericulture Program at the ‘Instituto Murciano de Investigación y Desarrollo Agrario y Alimentario (IMIDA)’, Murcia (Spain) (IMIDA coordinates: Long. 37.9388011, Lat. −1.1333683, Alt. 54 m.). The chemical characteristics of this extract were previously reported [10].

All reagents were provided by Sigma-Aldrich (Steinheim, Germany), unless otherwise stated.

### 2.2. Animals, Diets and Experimental Design

Male C57BL/6J mice (7–9 weeks old) were obtained from Charles River Laboratories (Lyon, France) and fed with either a standard chow diet (13% calories from fat, 20% calories from protein, and 67% calories from carbohydrates) (global diet 2014; Harlan Laboratories, Barcelona, Spain) or a high-fat diet with 60% of its caloric content derived from fat (purified diet 230 HF; Scientific Animal Food & Engineering, Augy, France). Mice were randomly distributed into four groups: control standard group (SD) (*n* = 8), control obese (HFD) (*n* = 8) and two obese groups treated with 250 mg/kg of metformin (MET) (*n* = 8) or 10 mg/kg of *M. alba* L. leaf extract (MAE) (*n* = 8) [10,20]. All the animals were housed in Makrolon cages, maintained in an air-conditioned atmosphere with a 12 h light/dark cycle and provided with free access to tap water and food.

Treatment administration was performed daily by oral gavage for 6 weeks. During this time, weight as well as food and water intakes were measured twice a week. Energy efficiency was calculated as the ratio between weight gain (g) and caloric intake (Kcal). Once mice were sacrificed, aorta, liver and epididymal and scapular brown fat were collected. Liver and fat weight indices were calculated by dividing their weights (g) by tibia length (cm). Samples were stored at −80 °C until further analysis.

All studies were carried out in accordance with the ‘Guide for the Care and Use of Laboratory Animals’ as promulgated by the National Institute of Health and the protocols approved by the Ethics Committee of Laboratory Animals of the University of Granada (Spain) (Ref. No. 28/03/2016/030 and 23/10/2019/174). The experimental procedures were performed in accordance with the ARRIVE guidelines [21,22].

### 2.3. Glucose Tolerance Test

A glucose tolerance test was carried out one week before the end of the experiment. Mice were fasted for 6 h and 2 g/kg of glucose solution was administered by intraperitoneal injection at the time of the test. Blood was collected from the tail vein at 0, 15, 30, 60 and 120 min after glucose administration. A handheld glucometer (Contour XT, Ascensia Diabetes Care, S.L., Barcelona, Spain) was used to determine glucose levels.

### 2.4. TBARS Assay

Liver and colonic lysates from mice were used to determine lipid oxidation by measuring the amount of thiobarbituric acid reactive substances (TBARS) [23]. Briefly, the malondialdehyde (MDA) resulting from lipid peroxidation reacts with the thiobarbituric acid (TBA) dissolved in dimethyl sulfoxide (DMSO), which was used in the extraction method. The products of the reaction are TBARS, a pink chromogen that was measured at 535 nm in a Magellan^®^ Tecan Infinite F50 spectrophotometer (Männedorf, Switzerland). TBARS levels were expressed as µM/mg protein in liver and colonic tissue.

### 2.5. NADPH Oxidase Activity

NADPH oxidase activity in aortic rings was determined by the lucigenin-enhanced chemiluminescence assay [24]. Aortic rings were placed for 30 min at 37 °C in HEPES-containing physiological salt solution (119 mM NaCl, 20 mM HEPES, 4.6 mM KCl, 1 mM MgSO_4_, 0.15 mM Na_2_HPO_4_, 0.4 mM KH_2_PO_4_, 1 mM NaHCO_3_, 1.2 mM CaCl_2_ and 5.5 mM glucose; pH = 7.4). Then, production of superoxide (O_2_^−^) by NADPH oxidases was stimulated by adding NADPH (100 M). Subsequently, lucigenin was injected automatically at a final concentration of 5 mol/L. NADPH oxidase activity was finally determined by measuring luminescence over 200 s in a scintillation counter (Lumat LB 9507, Berthold, Germany) in 5 s intervals and calculated by subtracting the basal values from those in the presence of NADPH. Activity was expressed as relative luminescence units (RLU)/min/mg of dry aortic ring.

### 2.6. Histological Studies

Histological studies were performed in samples from liver and epididymal adipose tissue. Sections were fixed in 4% paraformaldehyde (PFA) for 24 h. Depending on the application, tissues were processed in two different ways. Increasing concentrations of ethanol (70%, 85%, 95% and 100%) followed by paraffin embedding and trimming in 5 μm sections was carried out for hematoxylin and eosin staining. For oil red staining, the protocol consisted of increasing concentrations of sucrose (15% and 30%) followed by OCT (Tissue-Tek^®^ O.C.T. Compound, Sakura^®^ Finetek, Torence, CA, USA) embedding, frozen with isopentane at −40 °C and trimmed in a cryostat in 8 μm sections. Adipocyte size was measured in adipose tissue sections using Fiji imaging software with the Adiposoft v1.16 plugin.

Samples were evaluated by an independent pathologist.

### 2.7. Western Blot

Liver and adipose tissue were weighted and suspended in a lysis buffer (1:5 *w*/*v*) containing 20 mM of HEPES (pH 7.5), 10 mM EGTA, 40 mM β-glycerophosphate, 2.5 mM MgCl_2_, 1% Igepal^®^, protease inhibitors—1mM DTT, 2 μg/mL aprotinin (Cat.# A1153), 5 μg/mL leupeptin (Cat.# L9783), 1 mM phenylmethylsulfonyl Fluoride (PMSF) (Cat.# P7626), 1 μg/mL iodoacetamide (Cat.# I6125)—and phosphatase inhibitors—2 mM sodium orthovanadate (Cat.# S6508), 5 mM sodium fluoride (Cat.# S1504) and 1 mM sodium molybdate (Cat.# 331058). Amounts of 40–60 µg of protein were loaded into sodium dodecyl sulphate–polyacrylamide gel electrophoresis (SDS-PAGE, 6–12% polyacrylamide under reducing conditions) and electro-transferred to a PVDF membrane (Cat.# IPVH00010). Membranes were blocked with 3% BSA or 5% non-fat dry milk in PBS plus 0.1% Tween 20 according to the manufacturer’s instructions for each antibody (Table 1). Incubation with primary antibodies was performed overnight. Then, membranes were incubated with anti-mouse IgG (1:3000 dilution, Cat.# 7076 (Cell signaling)) and anti-rabbit IgG (1:5000 dilution, Cat.#A9169) HRP-conjugated secondary antibodies for 1 h of at room temperature. Finally, selected proteins were detected with the Western Lightning™ Chemiluminescence Reagent Plus (PerkinElmer Spain SL, Madrid, Spain). Signal acquisition was performed using a ChemiDoc image system (Bio-Rad, Hercules, CA, USA). Control of protein loading and transfer was conducted by detection of β-actin (ACTB) levels for cytoplasmic proteins. The quantification of bands was performed by densitometric analysis using ImageJ software v 1.53 (Free Software Foundation Inc., Boston, MA, USA).

### 2.8. Microbiota Analysis

Stool samples from each mouse were aseptically collected. Fecal DNA was isolated using the QIAamp PowerFecal Pro DNA Kit (Qiagen, Hilden, Germany) and region V3–V4 of 16S rRNA was amplified in accordance with the 16S Metagenomic Sequencing Library Preparation Illumina protocol. Sequencing was executed using the MiSeq 2 × 300 platform (Illumina Inc., San Diego, CA, USA). Bioinformatic analysis of gut microbiota samples was performed by using the QIIME2 software package (2021.11 version) and ‘R’ statistical software package (version 3.6.0; https://www.r-project.org/, accessed on 2 February 2023). Demultiplexed sequences were loaded into the program and quality control was performed [25]. Samples were then processed with DADA2 and amplicon sequence variants (ASVs) were obtained. The taxonomic assignment was calculated against the SILVA reference database [26]. Both Archaea and Eukaryota features were discarded from the analysis together with those features with a frequency lower than 10. The Phyloseq package was employed to determine alpha and beta diversity as well as relative abundance. For plotting the Venn diagram analysis and to represent both heatmaps and correlation plots, the Eulerr and MicroViz packages were employed, respectively (Larsson J (2022). Eulerr: Area-Proportional Euler and Venn Diagrams with Ellipses, R package version 7.0.0, https://CRAN.R-project.org/package=eulerr, accessed on 2 February 2023. Finally, DESeq2 was used to identify differences in taxa expression levels while a microbial package was used to assess possible biomarkers through linear discriminant analysis (LDA) effect size (LefSe) with an LDA score of 3 [27].

### 2.9. Statistical Analysis

Statistical analysis was performed using the GraphPad Prism version 7 software (GraphPad Software, Inc, San Diego, CA, USA) with statistical significance calculated at *p* < 0.05. All data are represented as mean (SEM) of at least 3 independent experiments/biological replicates unless otherwise stated in the figure legends. Multiple comparisons between groups were performed using the one-way ANOVA followed by Tukey’s test. When *p* < 0.05, different letters among groups were used and groups that share the same letter do not statistically differ. When no statistical significance among any of the compared groups was obtained, the graph showed no letter or symbol.

For the microbiota studies, statistical differences for alpha diversity and relative abundance were analyzed using the ANOVA test when there was a normal distribution or the Kruskal–Wallis test in case of a non-normal distribution. In contrast, beta diversity differences were measured through the Adonis function from the Vegan package.

## 3. Results

### 3.1. M. alba L. Extract Prevents Body Weight Gain, Reduces Fat Deposits and Improves Glucose Tolerance

**Figure 1:**
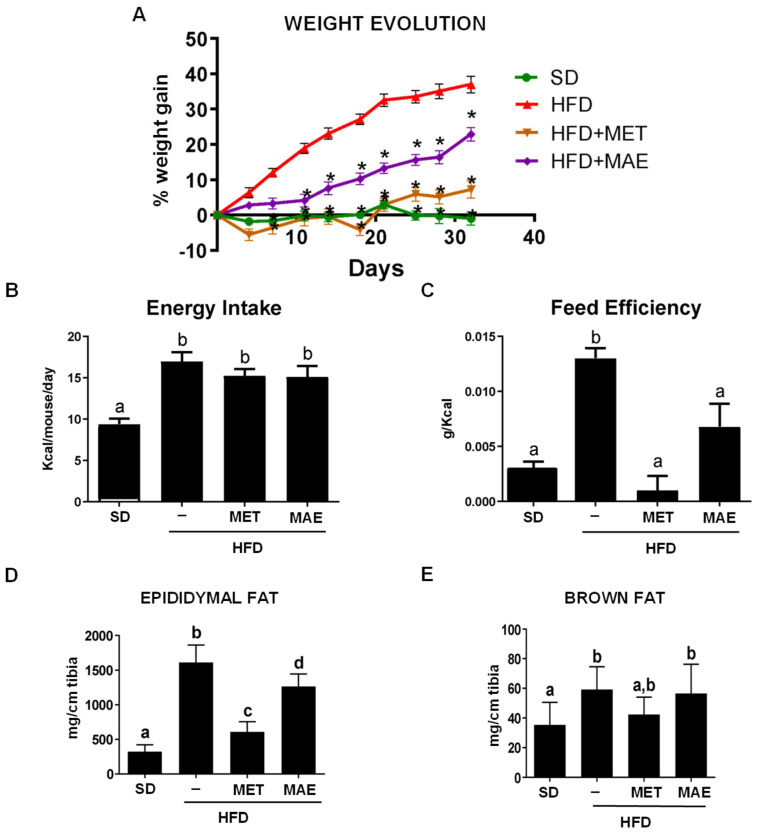
Mice treated with metformin (MET) or *Morus alba* L. extract (MAE). extract presented lower body weight gain as well as lower fat deposits without a satiating effect. (**A**) Body weight gain during the time of study. (**B**) Quantification of energy intake of the different groups of study. (**C**) Quantification of feed efficiency of different groups of study. (**D**) Total amount of epididymal fat. (**E**) Total amount of scapular brown fat. Data are expressed as means ± SEM (*n* = 8). * *p* < 0.05 vs. control HFD mice. Groups with different letters statistically differ (*p* < 0.05). SD: standard diet; HFD: high-fat diet.

In a previous study, different extracts from *M. alba* L. leaves collected from four genotypes (*Italia, Filipina, Kokuso* and *Valenciana Temprana*) showed anti-inflammatory and antioxidant properties in an experimental model of obesity in mice [10]. Among them, *Italia* genotype showed the best profile and it was selected to further investigate its beneficial effects against obesity, comparing them to those obtained with metformin, a drug currently used to manage type 2 diabetes. As shown in Figure 1A, a high-fat diet consumption resulted in an increase in body weight gain compared to those mice fed with a standard diet, which was significantly reduced by metformin. Similarly, *M. alba* L. leaves extract significantly reduced the increase in body weight from day 10 onwards. Interestingly, the reduction in body weight gain produced by both treatments was not associated with a satiating effect since no significant differences were observed when energy intake was evaluated in the different HFD-fed groups of mice (Figure 1B). Hence, both treatments induced a lower feed efficiency when compared to the control HFD-fed group (Figure 1C).

Accordingly, the deposits of epididymal and brown fat were significantly higher in the untreated HFD mice when compared to the SD group (Figure 1D,E). Both metformin and *M. alba* L. treatments exhibited a lower accumulation of epididymal fat deposits compared to the untreated mice fed a high-fat diet (Figure 1D).

**Figure 2:**
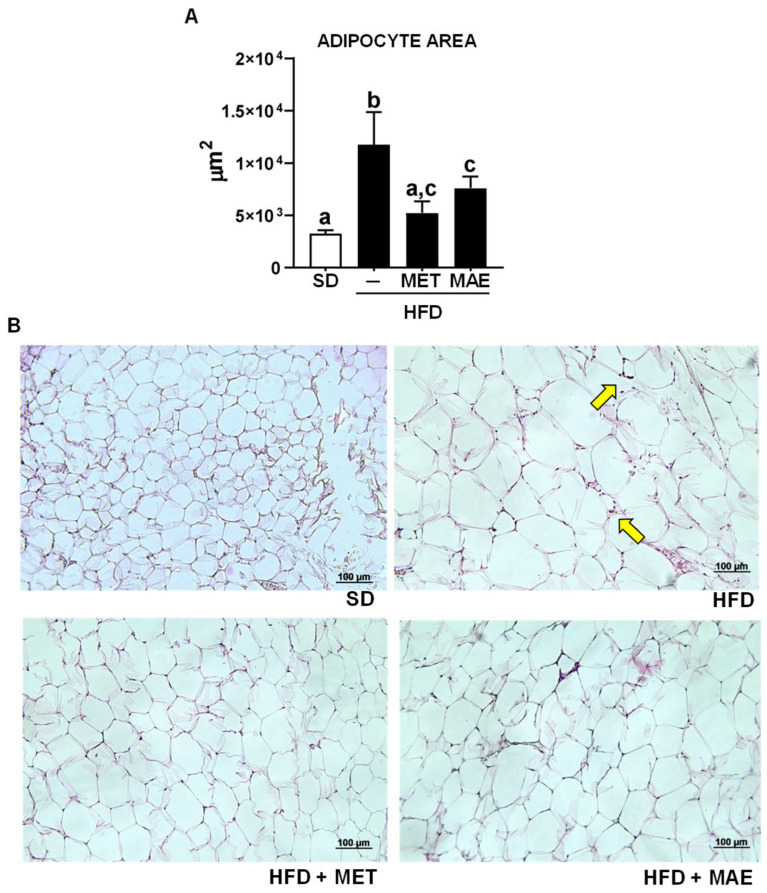
Mice fed with an HFD and treated with metformin (MET) or *Morus alba* L. extract (MAE) presented lower hypertrophy of adipocytes compared to HFD-untreated mice. (**A**) Adipocyte area quantification. (**B**) Representative images of adipocyte area of the different groups of study. Yellow arrows refer to the presence of immune cells. Data are expressed as means ± SEM (*n* = 8). Groups with different letters statistically differ (*p* < 0.05). SD: standard diet; HFD: high-fat diet.

It is well known that the increase in fat deposits is usually associated with adipocyte hypertrophy, typically evidenced by a significant increase in the epididymal adipocyte area in the untreated HFD-fed mice when compared with those fed a standard diet (Figure 2). More importantly, adipocyte hypertrophy was significantly reduced in those mice treated with metformin or the extract compared to untreated obese mice. In addition, the mice treated with either *M. alba* L. extract or metformin exhibited a lower infiltration of immune cells in epididymal adipose tissue sections when compared to the untreated HFD-fed mice (Figure 2B).

**Figure 3:**
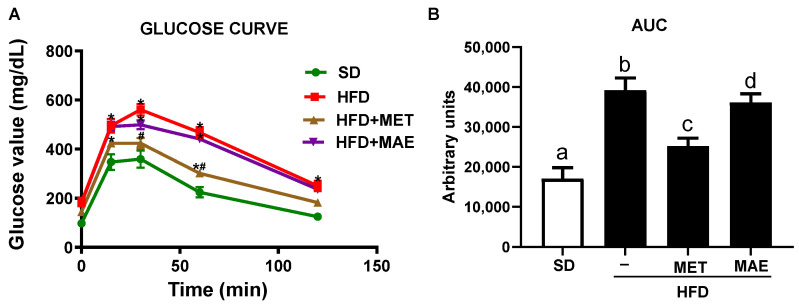
Mice fed with HFD and treated with metformin (MET) or *Morus alba* L. extract (MAE) presented lower blood glucose levels than untreated mice fed with the same diet. (**A**) Glucose curve representation of IPGTT. (**B**) Area under the curve representation of glucose curve. Data are expressed as means ±SEM (*n* = 8). * *p* < 0.05 vs. control SD mice and # *p* < 0.05 vs. control HFD mice. Groups with different letters statistically differ (*p* < 0.05). SD: standard diet; HFD: high-fat diet.

The impact of each treatment on glucose metabolism was also assessed in the glucose tolerance test. The results revealed that the HFD-fed mice showed a higher peak of the glycemic values 15 min after glucose injection compared to the control SD-fed mice, and these values remained increased at all time points that were evaluated (Figure 3A). As a result, a significant increase in the glycemic AUC in the control obese mice was observed (Figure 3B). As expected, metformin was able to significantly reduce the peak of glycemia and maintain glucose levels below those obtained in the control HFD-fed mice, leading to a significant reduction in glycemic AUC. Remarkably, the mice receiving the extract also showed lower plasma glucose concentrations at all time points compared to the untreated HFD-fed control mice, thus resulting in a significant reduction in the AUC (Figure 3A,B).

### 3.2. The Beneficial Effects of Morus alba L. Extract Were Associated with an Improvement in Obesity-Associated Inflammatory Status and Redox Homeostasis

**Figure 4:**
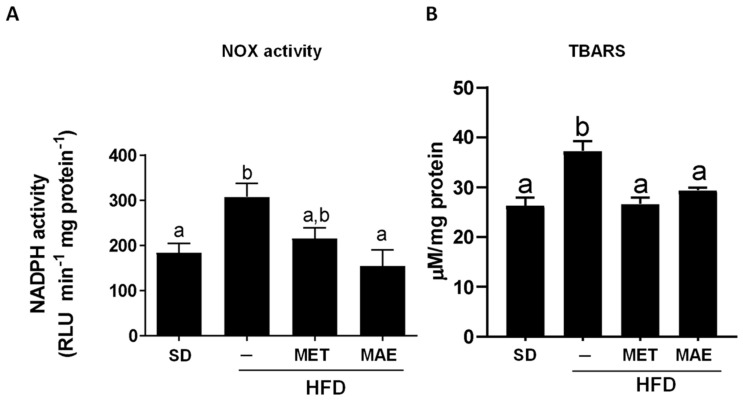
Metformin (MET) and *Morus alba* L. extract (MAE) treatments presented antioxidant properties in mice fed with an HFD by reducing NADPH oxidase (NOX) activity and TBARS levels. (**A**) NOX activity quantification in aortic rings. (**B**) TBARS total amount quantification. Data are expressed as means ±SEM (*n* = 8). Groups with different letters statistically differ (*p* < 0.05). SD: standard diet; HFD: high-fat diet; TBARS: thiobarbituric acid reactive substances.

The ability of *M. alba* L. extract to reduce weight gain and lipid accumulation, and to improve glucose metabolism, was related to an amelioration in the oxidative status derived from HFD intake. Thus, the antioxidant activity exerted by the treatments was evaluated by determining NADPH oxidase activity in aortic rings and TBARS levels in the liver. The results showed that the increase in NADPH oxidase activity and TBARS levels observed in the control HFD-fed mice was significantly reduced when the obese mice were treated with either *M. alba* L. extract or metformin (Figure 4).

**Figure 5:**
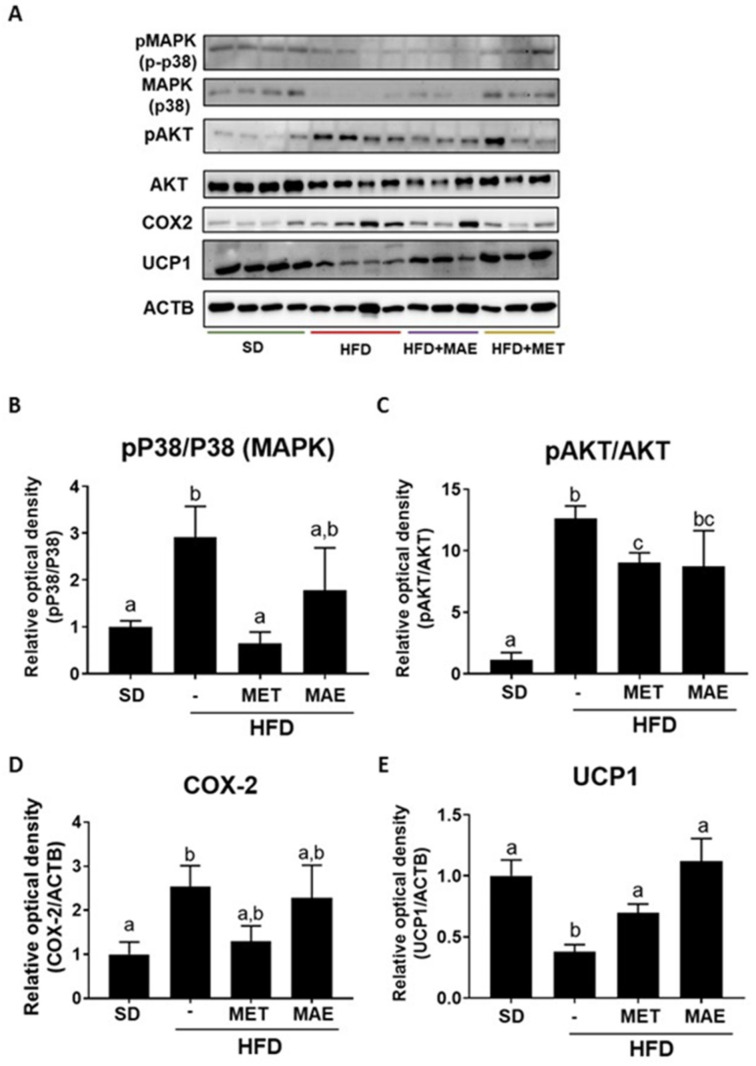
Metformin (MET) and *Morus alba* L. extract (MAE) treatments reduce the expression of inflammatory proteins in mice fed an HFD, suggesting an antioxidant activity. (**A**) Representative images of Western blot for each studied protein. (**B**) Western blot quantification for pP38 (MAPK). (**C**) Western blot quantification for pAKT. (**D**) Western blot quantification for COX2. (**E**) Western blot quantification for UCP1. Data are expressed as means ± SEM (*n* = 8). Groups with different letters statistically differ (*p* < 0.05). SD: standard diet; HFD: high-fat diet.

Since the obesity-associated oxidative stress contributes to the development of the characteristic subclinical inflammatory response, the expression of different proinflammatory markers was determined in fat and liver tissues. In epididymal fat, the expression levels of p38, AKT and COX-2 were determined, being these proteins closely related to the inflammatory response that occurs in obesity [28,29,30]. While metformin administration promoted a significant reduction in protein levels for MAPK and AKT, the extract showed a trend to reduce them, showing no statistical differences when compared to non-obese mice (Figure 5B,C). Similarly, untreated HFD-fed mice showed a significant increase in COX-2 expression compared to the SD-fed mice. Although both treatments showed no statistical differences in comparison with the obese control group, no differences were observed either when compared with the control lean mice (Figure 5D). Finally, the expression of mitochondrial uncoupling protein-1 (UCP1) was also evaluated in the fat tissue, which is the hallmark of brown adipocytes and is responsible for cold- and diet-induced thermogenesis, whose expression inversely correlates with inflammatory markers [31]. Interestingly, both treatments, *M. alba* L. extract and metformin, significantly restored the reduced UCP1 protein expression levels observed in the untreated HFD-fed mice (Figure 5E).

**Figure 6:**
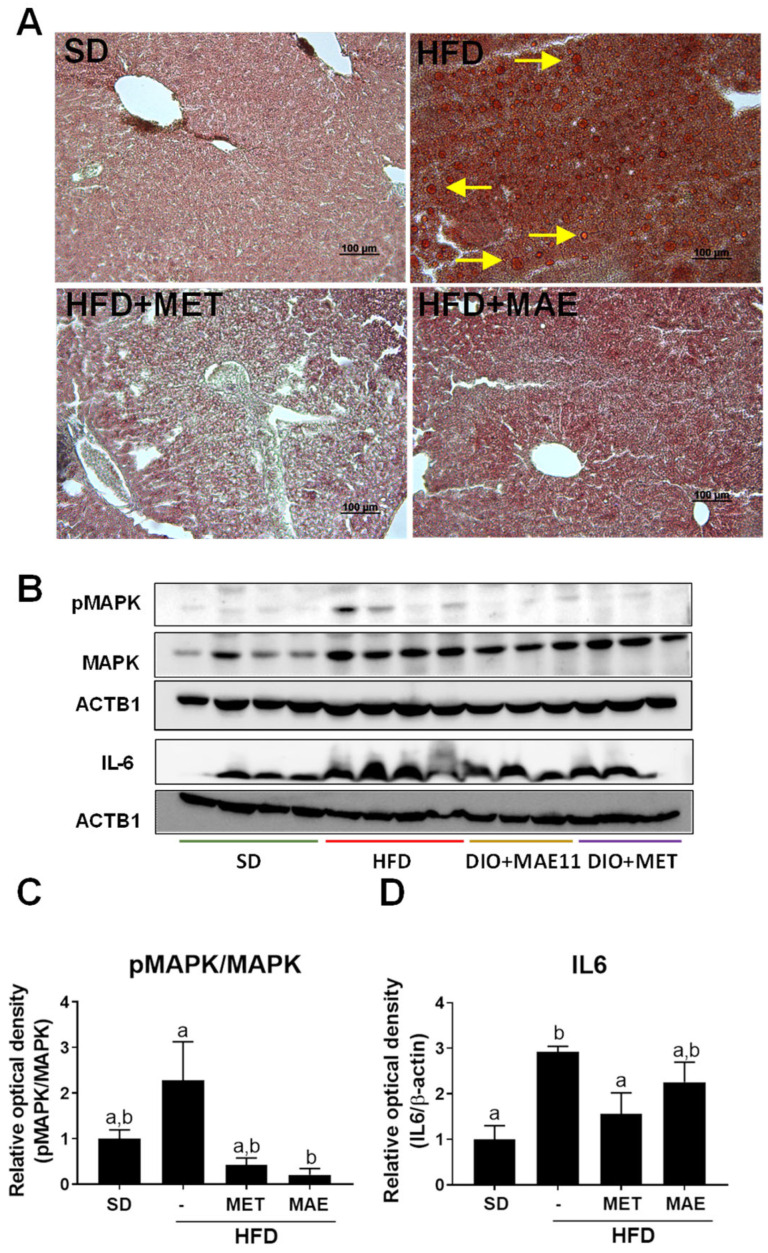
Metformin (MET) and *Morus alba* L. (MAE) treatments decreased lipid droplets in liver while attenuating p38-MAPK and IL6 expression in HFD-fed mice. (**A**) Histological images of liver status for each group of study. Yellow arrows refer to fat vacuoles. (**B**) Representative images of Western blot for each studied protein. (**C**) Western blot quantification for pP38 (MAPK). (**D**) Western blot quantification for IL-6. Data are expressed as means ±SEM (*n* = 8). Groups with different letters statistically differ (*p* < 0.05). SD: standard diet; HFD: high-fat diet.

The inflammatory status was evidenced in the liver by a higher expression of phosphorylated p38 MAPK and IL-6 in the HFD-fed mice in comparison with the non-obese control group (Figure 6B), which correlates with the hepatic steatosis and the inflammatory cell infiltration that took place in the liver as previously reported in obesity [32]. The administration of either the extract or metformin resulted in a significant reduction in the expression in both proteins, thus revealing an amelioration in the inflammatory response in this organ (Figure 6B). This beneficial effect on the liver was confirmed when the hepatic steatosis process was evaluated. Thus, HFD induced a significant accumulation of fat in the liver (macrovesicular steatosis), typically characterized by the presence of lipid droplets within hepatocytes. As expected, both treatments resulted in a significant reduction in hepatic steatosis, since hepatocytes mainly displayed a single small-sized lipid droplet or multiple lipid vesicles of very small size in their cytoplasm (Figure 6A).

### 3.3. Morus alba L. Extract Treatment Ameliorates Gut Dysbiosis in Obese Mice

**Figure 7:**
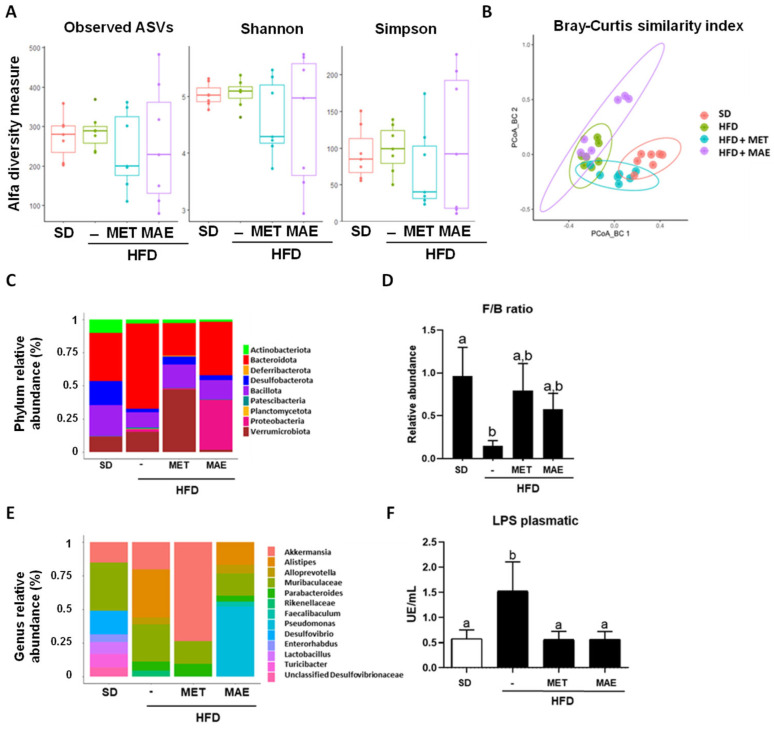
Analysis of gut microbiota composition revealed that treatment with metformin (MET) or *Morus alba* L. extract (MAE) restored dysbiosis produced by the HFD administration. (**A**) Alpha diversity index quantification. (**B**) Beta diversity index represented by Bray–Curtis distance. (**C**) Relative abundance at phylum level. (**D**) Quantification of F/B ratio. (**E**) Relative abundance at genus level. (**F**) Quantification of plasma LPS levels. Data are expressed as means ±SEM (*n* = 8). Groups with different letters statistically differ (*p* < 0.05). SD: standard diet; HFD: high-fat diet.

It is well known that obesity is associated with gut dysbiosis, and for this reason, in the present study, the impact exerted by *M. alba* L. leaf extract on the gut microbiota composition in HFD-induced obese mice was evaluated, and compared with that observed after the administration of metformin. When alpha diversity was determined, no significant differences were found among the experimental groups (Figure 7A). However, beta diversity analysis showed a clear separation among all groups (*p* < 0.001). Specifically, dissimilarity-based PCoA for *M. alba* L. extract presented significant clustering of the gut microbiota compared to all groups (*p* = 0.006) (Figure 7B). Next, abundance at phylum level was evaluated, and when the HFD-fed group was compared to the non-obese control, the control obese mice exhibited a notable increase in *Bacteroidota*, *Pseudomonadota* and *Verrucomicrobia* abundance and a significant decrease in *Actinomycetota*, *Bacillota*, *Desulfobacterota* abundance, thus resulting in a reduction in Firmicutes/Bacteroidota ratio (F/B ratio). Of note, the administration of *Morus alba* L. leaf extract or metformin to obese mice significantly modulated the phyla abundance; in fact, both treatments resulted in a bacterial profile more similar to the SD-fed group. Correspondingly, the F/B ratio was significantly ameliorated by both treatments (Figure 7C,D).

At the genus level, significant differences in the relative abundances of *Akkermansia*, *Alistipes*, *Alloprevotella*, *Parabacteroides*, *Desulfovibrio*, *Enterorhabdus*, *Lactobacillus* and *Turibacter* were observed (Figure 7E). Specifically, in the control HFD-fed group, *Alistipes* and Parabacteroides abundances were boosted whereas those involving *Desulfovibrio*, *Enterorhabdus*, *Lactobacillus* and *Turicibacter* were diminished compared to the non-obese control group. Interestingly, *M. alba* L. leaf extract administration was able to significantly reduce *Alistipes* compared to the HFD group, and remarkably, the plant extract treatment provoked an increase in two exclusive genera: *Faecalibaculum* and *Pseudomonas*.

In addition, plasmatic levels of LPS were determined as a marker of systemic endotoxemia associated with obesity. HFD intake results in increased intestinal permeability and systemic exposure to bacterial LPS. Thus, a metabolic endotoxemia is established which is derived from the dysbiosis status that occurs in obesity [33]. In fact, untreated HFD-fed mice showed higher levels of plasmatic LPS, while both treatments significantly attenuated this increment (Figure 7F).

### 3.4. Abundance of Bacteria Species Correlates with the Anti-Inflammatory and Antioxidant Response of Morus alba L. Extract

**Figure 8:**
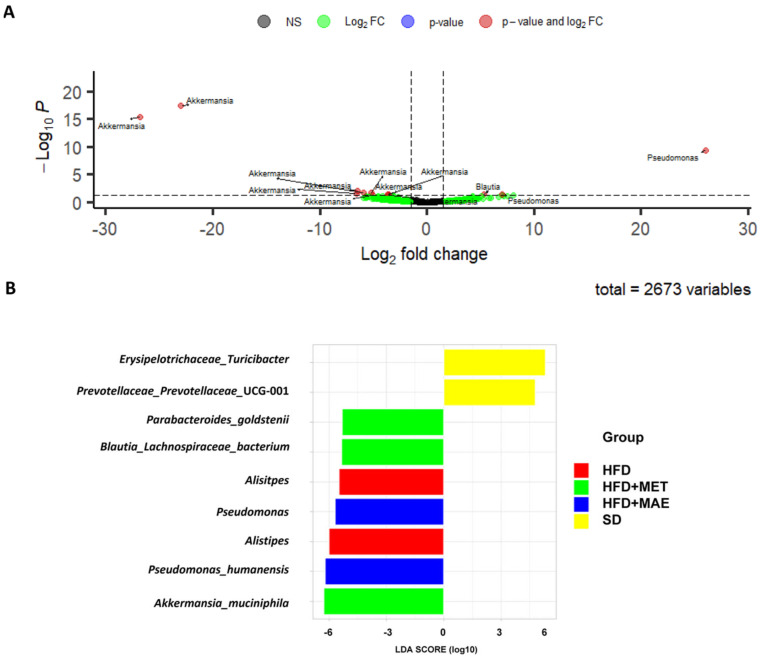
Volcano plot and LEfSe analysis showed specific bacteria increase depending on the administered treatment. *Morus alba* L. extract (MAE) presented a statistically significantly higher abundance of *Pseudomonas.* (**A**) Volcano plot showing differential abundance taxa of *Morus alba* L.-treated mice vs. metformin (MET)-treated mice. (**B**) Linear discriminant analysis (LDA) effect size (LEfSe) plot of taxonomic biomarkers (*p* value = 0.05 and LDA value = 3) (*n* = 8). SD: standard diet; HFD: high-fat diet.

The restoration of F/B ratio and reduction in LPS levels produced by both treatments suggests that some bacteria could be having a beneficial effect and restoring dysbiosis derived from HDF administration. A more specific analysis showed that extract treatment promoted a significant increase in Pseudomonas abundance (Figure 8A,B), whereas metformin was characterized by an increase in *Parabacteroides goldstenii*, *Lachnospiraceae* and *Akkermansia muciniphila*. In contrast, HFD was associated with a higher presence of *Alistipes* (Figure 8B).

**Figure 9:**
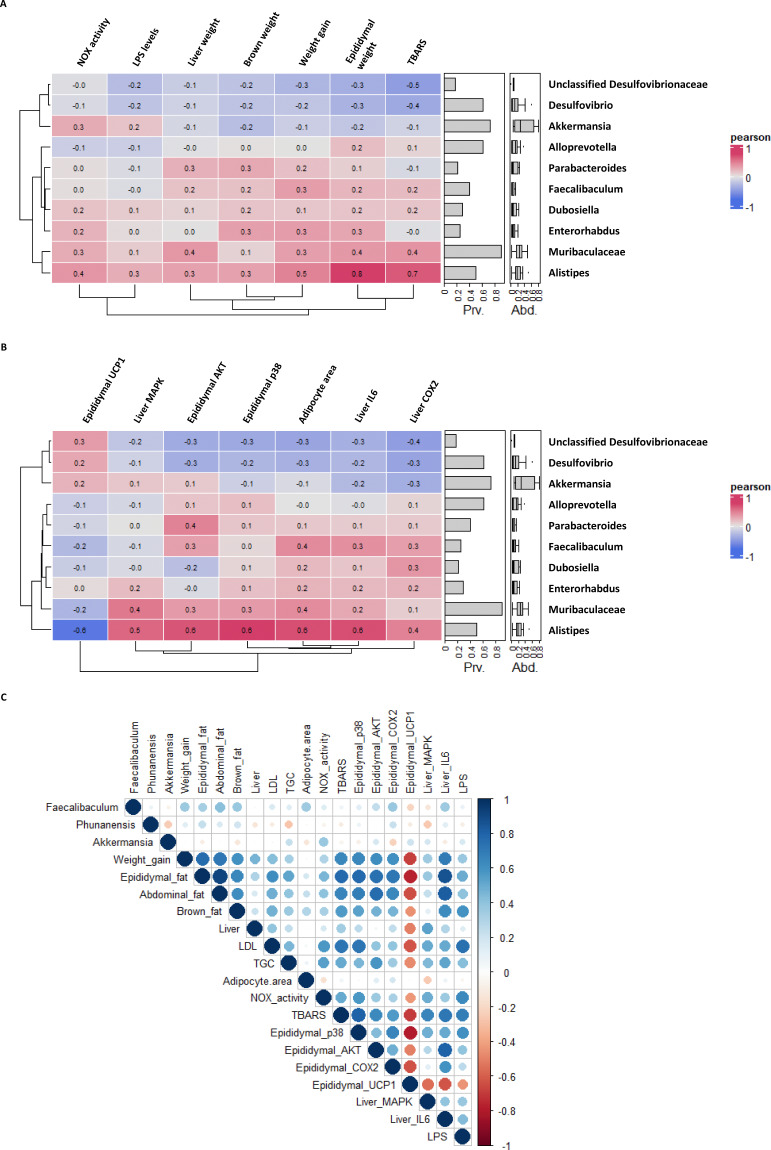
Significantly abundant bacteria found for untreated HFD, *Alistipes*, strongly correlated with the different inflammatory and oxidant markers analyzed. (**A**) Correlation plot for biochemical parameters evaluated during this study. (**B**) Correlation plot for studied protein levels. Blue colour meaning negative correlation; red, positive. (**C**) Correlation plot for specific bacteria of each group: *Faecalibaculum* and *P. hunanensis* (*Morus alba* L. extract—MAE); *Akkermansia* (Metformin). The Pearson correlation method was employed (*n* = 8). SD: standard diet; HFD: high-fat diet.

In order to analyze the influence of these specific bacteria, we performed different correlation studies. First, we observed the association between the most abundant bacteria of all groups with physiological parameters (Figure 9A) and protein levels (Figure 9B). In both cases, *Alistipes*, which was the most abundant bacteria of the HFD group, was strongly correlated with the studied variables. In addition, it showed a negative correlation for UCP1 levels, which was in accordance with the protein levels obtained in epididymal fat.

Subsequently, we decided to evaluate the effect of the taxa identified as significantly increased in both treatments. As it can be observed in Figure 9C, neither metformin (*Akkermansia*) nor *M. alba* L. extract (*Faecalibaculum* and *Pseudomonas hunanensis*) presented strong correlations with the analyzed variables, which correlates with the lower expression levels we have been observing along this work.

Compared to the associations of *Alistipes* previously described (HFD group), these results suggest that an increase or decrease in certain types of bacteria may have an effect over antioxidant and anti-inflammatory molecular pathways. As a result, *M. alba* L. extract effects may have been taking place through gut microbiota modifications that we have described.

## 4. Discussion

Lifestyle modification is the first line of action for the management of obesity. However, many obese patients do not achieve long-lasting benefits since it is difficult for them to adhere to the lifestyle changes due to their physical and psychological condition [34]. Drug therapy has been established as another way to address the problem of obesity. The most commonly used drugs approved by the U.S. Food and Drug Administration for the long-term treatment of obesity include orlistat, phentermine/topiramate, naltrexone/bupropion and liraglutide [35]. Unfortunately, the lack of efficacy of some of these drugs, which has been questioned in recent meta-analysis, or their high cost and frequent incidence of adverse effects have complicated their use [35]. Additionally, bariatric surgery yields substantial weight loss but its high cost and risk of serious complications make it only suitable for cases of severe obesity [34]. These findings have prompted the use of alternative therapies against obesity, highlighting the use of plant extracts with proven and safe anti-obesogenic properties [36,37]. In this scenario, *Morus alba* L. is a plant species containing different bioactive compounds, such as flavonoids and phenolic acids, that have previously demonstrated therapeutic potential against various diseases, including those metabolic disorders associated with obesity such as diabetes and dyslipidemia [38,39,40,41].

As expected from its described effects, the administration of *M. alba* L. extract to obese mice resulted in a significant reduction in body weight gain, which was accompanied by a reduction in the accumulation of fat deposits. These actions were exerted without affecting the satiety of mice, as evidenced by a similar energy intake among the different groups fed with HFD, thus discarding an anorexigenic action for these plant extracts. In consequence, other underlying mechanisms should be involved in the feed efficiency reduction observed. In the case of the liver, it has been reported that the excess of fat in obese individuals facilitates its accumulation and the impairment of its function. In fact, one of the main disorders associated with obesity is non-alcoholic fatty liver disease (NAFLD), which is characterized by hepatic steatosis [32]. In this context, *M. alba* L. administration revealed the improvement in liver steatosis as the lipid droplets observed in the tissue were lower than in mice fed with HFD. In addition, in both conditions, an increase in body weight gain along with greater lipid deposits and hepatic steatosis are usually linked to altered glucose metabolism [32,42]. This was confirmed in the present study, since HFD-induced obesity was associated with a worse sensitivity towards glucose while *M. alba* L. seemed to attenuate insulin resistance development. Although the impact of the plant extract on body weight gain was pronounced, this effect did not result in a marked amelioration of the glycemic profile evaluated by the glucose tolerance test. However, significant differences were found among groups when the areas under the glycemic curves were analyzed, thus suggesting an amelioration of the glycemic profile induced by the *Morus alba* L. treatment [43]. These findings are in concordance with our previous work [10], as well as other authors’ studies [43,44].

Two of the most important mechanisms involved in insulin resistance development and the other obesity-related pathologies are oxidative stress and inflammation [45]. Thus, *M. alba* L. could be modulating the derived effects of obesity by regulating these molecular mechanisms.

Oxidative stress is the result of an increase in reactive oxygen species (ROS) due to an imbalance in their production or degradation. As mentioned before, this increase is mainly due to a higher activity of NADPH oxidases, which are the main producers of ROS in the organism [46]. Hence, when NADPH oxidase activity was measured, *M. alba* L. extract displayed an antioxidant effect evidenced by a reduction in enzyme activity. Additionally, to confirm the antioxidant capacity of the extract, we evaluated TBARS levels. TBARS can be used for determining lipid peroxidation, a side effect of oxidative stress [47]. In accordance with NADPH oxidase activity, mice treated with *M. alba* L. presented a lower amount of TBARS, confirming the antioxidant capacity of this extract.

On the other hand, although oxidative stress promotes inflammation under obesity status [48], we further analyzed the anti-inflammatory properties of *Morus alba* L. Specifically, *M. alba* L. leaf extracts showed beneficial effects against the inflammatory status present in HFD-mice, evidenced by the ability of the extract to reduce the expression levels of COX-2 in epididymal fat and IL-6 in the liver. The activation of COX-2 in epididymal adipose tissue has been correlated with inflammation, insulin resistance and fatty liver in HFD-obese rats [49]. Moreover, HFD feeding is associated with an overexpression of IL-6 in the liver that contributes to the low-grade inflammation process observed in obesity [50].

Furthermore, we evaluated the expression levels of some genes that are related to the inflammatory molecular pathway and could explain the lower weight gain, lipid accumulation and better glucose uptake produced by the plant extract. In a postprandial physiological situation, the increase in blood glucose stimulates the release of insulin from the pancreas, which reaches different target tissues and binds to its receptor. This action triggers the activation of the PI3K/AKT cascade, in which phospho-AKT promotes the translocation of the glucose transporter (GLUT) to the plasma membrane, thus inducing the entry of glucose into the cell [51]. In adipose tissue, the activation of this signaling cascade results in the promotion of lipid biosynthesis and the suppression of lipolysis. Therefore, activated AKT contributes to an increase in fat deposits [29]. Additionally, it has been reported that insulin resistance may result in overactivation of the PI3K/AKT pathway [52]. This would justify that, in obese mice, the high and sustained levels of blood glucose would result in the activation of AKT, in comparison with lean mice. Moreover, this action may be involved in the increased fat accumulation observed in untreated obese mice. Interestingly, a reduction in the activation of AKT was observed in those obese mice treated with metformin or *M. alba* L. extract, which could be associated with the observed amelioration of the glucidic metabolism profile and the substantial reduction in fat deposits.

Moreover, thermogenesis could also explain the effects derived from *M. alba* L. administration. Specifically, UCP1 protein is considered a major regulator of adipose energy expenditure and metabolic homeostasis through thermogenesis activation [53,54]. UCP1 uncouples the proton motive force generated in the mitochondria from ATP production to produce heat, thus increasing the energy expenditure. A deficiency of this protein in mice is associated with increased weight gain and the development of obesity. Conversely, an overexpression of UCP1 is associated with an anti-obesity effect [55]. Of note, *M. alba* L. extract was able to significantly increase the UCP1 levels downregulated in obese mice, and this may be considered one of the main mechanisms involved in the anti-obesity effects displayed by this plant extract.

As previously described, another accepted hypothesis to explain the systemic inflammatory state linked to a fat diet lies in the translocation of bacterial components such as LPS that causes metabolic endotoxemia [56]. LPS leakage is associated with gut dysbiosis and an impairment of the gut barrier integrity after high-fat exposure [56,57]. Moreover, gut dysbiosis associated with obesity results in the release of LPS by Gram-negative bacteria that diffuses through the altered tight junctions or is incorporated into chylomicrons [58]. These findings were confirmed in the present study, in which an increased level of plasmatic LPS was found in control mice fed an HFD compared to those fed a normal diet. Interestingly, this metabolic endotoxemia was reduced in those groups of mice treated with the two treatments evaluated: *M. alba* L. extract and metformin.

Additionally, gut microbiota composition was also studied as an extract from a Chinese *M. alba* L. variant had already been described as a functional food capable of improving gut dysbiosis. Bacterial alpha diversity was not significantly modified in the mulberry evaluation assay but there was a tendency for treatments to increase richness and evenness. Despite this, gut dysbiosis produced by the HFD intake was confirmed when the beta diversity was determined. The different clusters observed in the PCA analysis suggest that each condition was characterized by the presence of different bacteria rather than differences in abundance within the ones that are shared among groups. Determination of these specific bacteria could be useful to identify some particular species that could prevent obesity-derived pathologies, considering that these results suggest that the HFD impacts the bacterial microorganisms involved in the efficacy of energy harvesting [59,60,61]. Both treatments, metformin and *M. alba* L. extract, were associated with a modulation of gut dysbiosis. When the obese mice were treated, a normalization of the levels of *Bacteroidota* and *Bacillota* phyla was found. Changes in bacteria genera were also observed in the obese mice compared to the lean mice. *M. alba* L. extract significantly modulated many of them. Remarkably, a reduction in *Alistipes* genus abundance was observed in the plant extract-treated group. *Alistipes* may have protective effects against some diseases, including liver fibrosis, colitis, cancer immunotherapy and cardiovascular disease. However, several reports have indicated that *Alistipes* is pathogenic in CRC and is associated with gut inflammation [62]. In our study, the increase in *Alistipes* in untreated mice fed with an HFD also correlated with a higher expression of inflammatory cytokines and lipid accumulation, suggesting that the reduction in its abundance promoted by *M. alba* L. extract could have a beneficial effect. Additionally, the extract treatment increased *Faecalibaculum* content, which was reduced in the untreated HFD-fed mice. These are Gram-positive obligate anaerobes with high fermentation ability, very efficient in producing butyrate, which have been associated with the SCFA production and carbohydrate metabolism. Consequently, the amelioration of the obesity-associated dysbiosis in *M. alba* L. extract-treated obese mice may contribute to the beneficial effects observed in this experimental model of diet-induced obesity.

## 5. Conclusions

The effects displayed by *Morus alba* L. leaf extract in this experimental model of high-fat diet-induced obesity in mice provide convincing evidence supporting its anti-obesity activity and the capacity to ameliorate the obesity-associated metabolic disorders. This study highlights the prebiotic effect of the *M. alba* L. leaf extract and associates it with its antioxidant, anti-inflammatory and anti-obesogenic properties in a murine model of high-fat diet-induced obesity. Therefore, this extract can be considered as a promising candidate for the treatment of obesity, as well as in the prevention of its associated conditions, including cardiovascular diseases and type 2 diabetes.

However, our study has some limitations, the most important being that it has been carried out in a preclinical murine model; therefore, our findings cannot be directly transferred to humans. This means that intervention studies in humans, which are in progress in our research group, are necessary.

## Figures and Tables

**Table 1:** Primary antibodies used for Western blot.

Antibody	Reference	Company
Anti-phospho MAPK(p38) Anti-MAPK(p38)	9211 9212	Cell signaling Cell signaling
Anti-UCP1	sc-293418	Santa Cruz
Anti-COX2	12282	Cell signaling
Anti-phospho-AKT	9271	Cell signaling
Anti-AKT	9272	Cell signaling
Anti-IL6	12912	Cell signaling

## Data Availability

Data is contained within the article.
